# Abstract of the Proceedings of the Buffalo Medical Association

**Published:** 1861-10

**Authors:** J. F. Miner

**Affiliations:** Secretary


ART. II.—*Abstract of the Proceedings of the Buffalo Medical Association*

TUESDAY EVENING, Sept. 3d, 1861.

Association met at the usual hour. Present—Dr. Gay, President, in the Chair, Drs. Eastman, Samo, Gould, Congar, Miner, Cronyn, Ring.

*The Secretary* asked the Association to amend or accept the Minutes of the last meeting, as published in the *Medical and Surgical Journal*, without being read, and remarked: "As Editor of the *Medical Journal*, I have a new and increased interest in the proceedings of this Association. I have always looked to it as to the "time dial" of the profession in Buffalo, and believe it has indicated truly the point of progress. In receiving many pleasant words of approval and encouragement, in my new enterprise, from the profession in all parts of the country, it is remarkable how much importance is attached to the Transactions of the Buffalo Medical Association. It is often estimated worth the price of "fifty such Journals" to again receive the report of the proceedings of this Association.—My purpose in telling you this, is to stimulate your efforts, so that your present reputation may be sustained, and to say that it *will not be sustained* without the most earnest endeavor. This good name has fallen to us rightfully, and we hold it in common, by virtue of being found in good company. It is the *living, active, earnest, progressive* men of the profession, who have earned it for us, and we are in no way indebted for it, to those who cannot be induced to become members of our Association. When judged in this matter by our peers, outside the circle of local jealousies, we receive the universal verdict of approval. In my efforts to collect what is truly valuable, and abridge what may not be of general interest, I ask your indulgence and co-operation; I have also to request that as much as possible communications be written, rather than verbal; it generally will insure greater accuracy and increased interest, and confer upon me personally a great favor, if correctly prepared for the press. If from any cause this may not be, I have to ask, that verbal communications be reported for me by the author, and sent as soon as possible to my office. You are all equally interested with me, and you will, I trust, excuse me for presenting this matter at this time."

The Minutes of the last meeting were approved as published in the *Medical and Surgical Journal.*

Dr. C. P. Fanner was elected member of the Association on compliance with the By-Laws. Action on the proposition for membership by Dr. Sweet, was postponed on account of his removal to New York City.

*Dr. Miner* read the following paper:

Within the last few months I have treated several cases of enlarged bursæ. These tumors have been situated in different locations, been treated by different methods, and attended by variable results. I have thought that this Association might be interested in a *resume* of the history of some of these cases, with the results attending the different modes of treatment.

Visited I. S. with inflamed bursæ over the patella of two years standing; had been treated by several physicians, but mostly by Homœpathic practitioners; had suffered at times considerably, and been prevented from usual business; knew of no cause for the trouble unless a slight injury received in shutting a drawer by pressing with his knee. Inflammation was very great, and I was called, since amputation was thought likely to be required, and the practitioner usually in attendance used tinc. arnica, "*did not do surgery*." This case I punctured, and drawing off about two ounces of serum, injected tinc. iodine into the sac; five days later, serum still continuing to escape, considerable fluctuation remaining, I opened the sac through its entire length, placed lint in the wound and cavity, and applied poultice. The pain was not severe, healthy granulations soon appeared, and in about ten days it was wholly closed.

The second case I will mention was also over the patella. This patient was a truckman; his trouble was of two weeks duration; knew of no injury acting as a cause; it was highly inflamed, greatly swollen, and very painful. This I opened through its entire length, a distance of three inches or more ; a large quantity of pus, and a synovial like fluid in about equal proportions escaped, coming up so freely from the sides of the leg, that I was fearful I was dealing with purulent formation from the knee joint. The previous pain, sleeplessness, loss of appetite, and extensive discharge greatly reduced the strength of the patient, making him look pale, haggard and anxious, with expression of severe disease. Under the influence of tonics and nutritious diet, healthy action in the part soon commenced, and in three weeks from the time of operation, the wound had healed, and he was dismissed from farther attendance, rapidly improving in flesh and strength.

*Case Third.*—J. M. advised to consult me by Dr. Gould of this city, who had the foresight to decline the treatment. The tumor was situated over the radio capal articulation upon the posterior portion of the wrist. Patient thought it was caused by severe straining of the wrist in working at his trade of cabinet maker. He had been under treatment by various physicians, mostly by plasters, compression, liniments, &c. I attempted to break the sac sub-cutaneously by striking the prominence with a book quite sharply, while the wrist was strongly flexed. Failing in this very inelegant operation, and my patient remaining very resolute to have something done to relieve the difficulty, I commenced incisions with the view of dissecting out the whole sack, in this operation I was also only partially successful, it was so strongly and extensively attached to the extensor tendons that complete removal was impossible ; removing what could be, I applied simple dressing, hoping it would heal as a common flesh wound usually does.—The external portion of the incision healed over and appeared as if all was completely well; the fifth night he had chill, severe pain, and re-accumulation of fluid; an opening through the newly formed cicatrix allowed the escape of a synovial like fluid with immediate relief of the pain and swelling; iodine tinc. was injected, and linen pledgets dipped in it, introduced deeply into the cavity ; when nothing was kept in the opening to prevent union, this appearance of healing would be repeated, followed by pain, re-accumulation of fluid, &c., requiring to be opened, the same fluid escaping. I now opened the tumor through the whole length, painting the surface freely with tinc. iodine, lint dipped in iodine was placed in the opening, but as yet the same fluid seemed to secrete and discharge as plentifully as ever. With the view of exposing the surface beneath as much as possible, I now made free incision in the opposite direction; at length we had a somewhat formidable looking case, luxuriant granulations springing up in some parts, standing out prominently, and from the deeper surfaces synovial-like fluid escaping very abundantly; iodine, nit. silver, poultices and *time*, were all faithfully used. This case after giving us plenty of trouble, healed down nicely, so that at the end of the tenth week he was able to resume his usual occupation.

*Bursæ in the Popliteal space.* I desire to speak briefly of this case, mostly on account of its location, though possessing interest in common with other cases of this form of disease, on account of the results of treatment.

J— P— consulted me a few weeks since on account of a most violent pain in the hip joint, extending down the leg, so severe as to prevent all business, sleep or comfort, and at times absolutely intolerable; he had also in the ham a tumor which had been noticed for several years, gradually increasing in size, but never producing much inconvenience. He had been under the care of a worthy physician for the relief of the pain, which was regarded as rheumatic in character. It being very intense, and in no way relieved, the tumor being present, it was feared that some connection existed between the tumor and pain, and with this view the patient was advised to ask my attendance. Six grs. sul. quinine every six hours sufficed to remove all pain in two days' time, showing its neuralgic or malarial character.

The tumor next claimed attention, as the patient was not satisfied until relieved of that also. Dr. Eastman being in my office was kind enough to examine the case with me, and participated in the opinion that it was probably a fatty tumor. We had considered the fact that in this location we might have popliteal anurism, solid tumors, fibrous, encephaloid, and fatty. From the absence of pulsation we excluded the possibility of anurism, and to my proposition to remove, by dissecting it out, consent was given by all parties interested. Cutting down I found distinct thick fibrous cyst, and could discover by the clear color and fluctuation that it contained watery fluid; it extended four or five inches, occupying the whole popliteal space; was attached to the tendons so extensively and closely that to remove it entirely, was wholly impossible. Upon opening the sac about eight ounces of synovial-like fluid escaped. I now wished myself and patient out of the trouble which I anticipated might follow. Painting very thoroughly the large surface now exposed, after removal of what of the cyst could be removed, with tinc. iodine, and placing a pledget of linen in the opening, to prevent early union of the integument, I directed a large poultice to be applied and perfect rest to be observed; the pain, described as a "live coal of fire in the wound," to be allayed with sufficient quantity of opium. This healed as kindly and rapidly as such incisions usually would in healthy tissue.

I have notes of several other cases, but these will serve as representatives of the class. I have often attempted the cure of these affections by introduction of seaton; my success has not been signal, and generally before complete recovery took place, I have removed the seaton and made the free opening.

I do not speak of bursal tumors for the purpose of giving any new views upon the subject, or supposing that my experience is greatly unlike that of others who attempt their removal. I have related these cases hoping the treatment described, and its results may contribute somewhat as a guide, until riper experience shall have dictated a wiser plan, and also expecting to elicit any views and opinions which may be entertained by the members of the Association, giving any instruction as to the character or treatment of these very troublesome affections.

*Prof. Eastman* said he had been greatly interested in the cases related by Dr. Miner since the different modes of treatment he had adopted, and the results following each plan, had been so clearly and conclusively shown. His own experience had not been very extensive, yet sufficiently so to convince him of the great interest and importance, which properly attaches itself to the treatment of enlarged Bursæ. In the cases he had treated he had been very successful, but had not now in recollection anything so extensive and severe as those described by Dr. Miner. The pathology of Bursal tumors involved a wide field for investigation. Bursæ constitute the simplest form of the synovial system; are developed beneath the skin where it is exposed to unusual and permanent pressure and friction. as in club-foot, over the joint of the large toe, or on the stump of an amputated limb; they are observed in deeper parts between muscles and tendons, as well as between them, and the unusual protuberances of bone; dislocated bones are sometimes firmly fixed by a new synovial capsule; and the false joints formed in cases of ununited fracture, furnish another example of this same new growth. Was not prepared to speak at much length, not knowing that this subject was to be brought up for consideration; desired to thank the Secretary for the report he had presented, and for the example, so worthy of imitation, he had given the Association.

*Dr. William Ring* reported a case of *re-dislocation of the Humerus.* Mrs. V— R—, aged 33, a strong and muscular woman fell and dislocated the humerus downwards, or into the axillal, on the 21st of August. I made three unsuccessful efforts at reduction with the heel in the axilla. In the last was assisted by Dr. T., who had also been sent for when the accident first happened. Chloroform was now administered, the patient being fully brought under its influence, when we succeeded in making the reduction at the first attempt, the bone returning with an audible snap, the motions of the arm and the rotundity of the shoulder being restored. Dr. T. then left.

While I was preparing a bandage, in less than ten minutes after the reduction, and before consciousness had fully returned, the patient raised up, unnoticed by me, on the elbow of the same side, and vomited, and then relapsed back on the floor. When my attention was next turned to her, I noticed the arm again thrown from the side and a flattening under the acromion, and soon found by examination the head of the bone in its former position. A little more of the anesthetic was given, and I again made the reduction, the bone returning as before. The dressing was made in the usual manner, with a small pad, bandage, and sling, which after ten days were taken off at the request of the patient. Except a little lameness it is now well, and the motions perfect. Less care, and inability to account for its being out of joint, would certainly have made me liable to action for mal-practice, resulting perhaps in a heavy judgment against me.

*Dr. Miner* said that in such cases re-dislocation was to be feared and guarded against; the wearied muscles were not in condition to hold it firmly in place; added to this the relaxation from chloroform, would also very much aid in such a result; did not look upon the occurrence of re-dislocation as unusual, if left without constant and firm support until permanent dressings could be applied.

*Prof. Eastman* regarded the perfect motion and use of the arm in three weeks' time, as related by Dr. Ring, a remarkable result. In all cases he had attended, a much longer period elapsed before anything like perfect use was obtained. Wasting of the deltoid muscle has also been a common result in the cases seen by him. Dr. E. related a case of great wasting of the deltoid muscle where every effort was made to prevent it. His attention had been early called to this point by treating a very troublesome case when he first opened his office as a physician. The wasting of the muscles is clearly due to injury of the nerves; if from the dressings and want of use, why not this condition after fracture of the fore-arm, which requires dressing and rest for a longer time?

*Dr. Gould* inquired how long a dislocated arm required dressing, and related a case of dislocation of the humerus in a student of medicine who had immediate use and had no dressings whatever. Also the case of an Irishman whose humerus was dislocated; chloroform given, and reduction easily effected, and dressings were applied in the usual manner. The motions of the joint seemed fully restored immediately after the reduction Has seen Dr. G. reduce a dislocated humerus and let it go without dressing, and thinks the case done well. Does not advocate or defend that plan of treatment, but thinks some cases would do well even thus neglected, while others apparently not worse injured or displaced, would require attention for a long time, and perhaps never regain full strength and motion.

*Prof. Eastman* remarked, that he had no recollection of any books or author, who did not recommend dressing, rest, &c., after dislocation. Books and teachers speak also of the importance of sustaining the arm until permanent dressings can be applied, and the dangers attending the neglect of so doing.

*Prevailing Diseases.*—Cholera Morbus, Diarrhœa, Dysentery, Mumps Scarlet Fever, Whooping Cough, Diphtheria and Typhoid Fever, were reported as occurring in the practice of the physicians present.

*Dr. Gay*, in taking charge of the soldiers stationed at Fort Porter, had met 20 cases diarrhœa the 2d day of September, 30 cases the 3d day; this was attributed to the free use of very dark sugar, by some, others thought that the water used was the cause of the disease.

*Dr. Samo* remarked, that though the physicia nspresent had expressed their sense of bereavement on account of the death of Dr. Treat, in their action in the Erie County Medical Society, yet it would seem not inappropriate that suitable expression be also made in this Association. Dr. Treat has been one of the most active, energetic and worthy members, always present at the regular meetings, ready to communicate from the treasures of his well stored mind; every one must have felt with me the deepest sadness in observing this evening his vacant chair, though perhaps no one can fully appreciate my feelings since Dr. Treat and myself occupied this office in company—"the one is taken and the other left."

*Dr. Gould* said, he hoped that the sentiments of high respect entertained by this Association, for the worth and memory of Dr. TREAT, would be properly expressed and left upon our records, that those who come after us may see some tokens of our appreciation of the character and ability of Dr. Treat; and would therefore move that a committee of three be appointed to present resolutions, &c.

Drs. Gould, Samo and Ring, Committee, presented the following resolutions, which were unanimously adopted :

*Inasmuch* as by a stroke of an All Wise Providence, at once sudden, inscrutable and afflictive, our professional brother Dr. Wm. Treat has been called away from our side and from his earthly labor, therefore, as a feeble expression of the sense of this Association,

*Resolved,* That in the death of Dr. Treat, our Society deplores the loss of one who was ever ready to discharge faithfully and fully his share of its duties and responsibilities—our profession, the loss of one who possessed in rare combination scholarly taste and purpose, with practical tact and skill, and a conscientious and self-forgetting devotion to the well-being of those entrusted to his care—and Society at large, the loss of one of its most guileless, unselfish and intelligent citizens.

*Resolved,* That great as is our grief at his departure, we feel it to be trivial compared to the irreparable loss of those near and dear to him in the sanctuary of home ; and while we tenderly commend them to the God of the widow and the Father of the fatherless for true solace ; we do hereby also tender to the stricken widow and family an expression of our sincere and profound sympathy, impotent to relieve though it be, in this their dark hour.

Special subject for the next meeting—"*Relation between teething and diarrhœa.*" Adjourned.

J. F. MINER, Secretary.

